# Methyl Jasmonate and Sodium Nitroprusside Jointly Alleviate Cadmium Toxicity in Wheat (*Triticum aestivum* L.) Plants by Modifying Nitrogen Metabolism, Cadmium Detoxification, and AsA–GSH Cycle

**DOI:** 10.3389/fpls.2021.654780

**Published:** 2021-08-05

**Authors:** Cengiz Kaya, Ferhat Ugurlar, Muhammad Ashraf, Ahmed Noureldeen, Hadeer Darwish, Parvaiz Ahmad

**Affiliations:** ^1^Soil Science and Plant Nutrition Department, Harran University, Sanliurfa, Turkey; ^2^Department of Botany, University of Agriculture, Faisalabad, Pakistan; ^3^Department of Biology, College of Sciences, Taif University, Taif, Saudi Arabia; ^4^Department of Biotechnology, College of Sciences, Taif University, Taif, Saudi Arabia; ^5^Department of Botany, S.P. College Srinagar, Jammu and Kashmir, India

**Keywords:** cadmium toxicity, inorganic nutrients, methyl jasmonate, wheat, oxidative stress

## Abstract

The principal intent of the investigation was to examine the influence of joint application of methyl jasmonate (MeJA, 10 μM) and a nitric oxide–donor sodium nitroprusside (SNP, 100 μM) to wheat plants grown under cadmium (Cd as CdCl_2_, 100 μM) stress. Cd stress suppressed plant growth, chlorophylls (*Chl*), and PSII maximum efficiency (*F*_*v*_/*F*_*m*_), but it elevated leaf and root Cd, and contents of leaf proline, phytochelatins, malondialdehyde, and hydrogen peroxide, as well as the activity of lipoxygenase. MeJA and SNP applied jointly or singly improved the concentrations of key antioxidant biomolecules, e.g., reduced glutathione and ascorbic acid and the activities of the key oxidative defense system enzymes such as catalase, superoxide dismutase, dehydroascorbate reductase, glutathione *S*-transferase, and glutathione reductase. Exogenously applied MeJA and SNP jointly or singly also improved nitrogen metabolism by activating the activities of glutamine synthetase, glutamate synthase, and nitrate and nitrite reductases. Compared with individual application of MeJA or SNP, the combined application of both showed better effect in terms of improving plant growth and key metabolic processes and reducing tissue Cd content, suggesting a putative interactive role of both compounds in alleviating Cd toxicity in wheat plants.

**Main findings:**

The main findings are that exogenous application of methyl jasmonate and nitric oxide–donor sodium nitroprusside alleviated the cadmium (Cd)–induced adverse effects on growth of wheat plants grown under Cd by modulating key physiological processes and up-regulating enzymatic antioxidants and the ascorbic acid–glutathione cycle–related enzymes.

## Introduction

Cadmium (Cd) is not required by plants for their optimum growth, so a slight increase in its levels in a growth medium causes considerable damages to several metabolic processes (Shanmugaraj et al., 2019), such as limitation of assimilation rate (Anwar et al., 2019; Zhou et al., 2020) and disturbance in plant water balance (Naeem et al., 2019). Moreover, Cd stress causes overproduction of reactive oxygen species (ROS) in plants (Gallego and Benavides, 2019). Overaccumulation of ROS can considerably damage the key membrane biomolecules, thereby causing leakage of all biological membranes (Jia et al., 2020). Plants possess a well-developed antioxidant defense system to counteract multiple stresses including Cd stress (Zaid et al., 2019). For example, one such promising mechanism is the modulation of the ascorbic acid (AsA)–glutathione (GSH) cycle enzymes (Khan et al., 2019). However, such a protective strategy does not constantly work in most plant species including wheat under Cd stress, because of being the crop highly sensitive to this metal stress (Rizwan et al., 2016).

Nitrogen (N) metabolism is a key physiological event that substantially affects growth, yield, and quality of most plants (Singh et al., 2016; Rajwade et al., 2018). Plants absorb N basically in the form of nitrate (NO_3_^–^) by roots and transfer it to leaves for its assimilation therein (Xuan et al., 2017). Nitrate reductase (NR) carries out the reduction of NO_3_^–^ to NO_2_^–^ (nitrite), and then nitrite reductase (NiR) converts NO_2_^–^ to ammonium (NH_4_^+^) (Kaiser et al., 2018). The conversion of NH_4_^+^ to glutamate and glutamine takes place through glutamate synthase (GOGAT) and glutamine synthetase (GS) (Lea and Miflin, 2018). Thus, it is critically important to maintain optimal the activities of the N metabolism–related enzymes, i.e., NR, NiR, GS, and GOGAT, for maintaining optimum growth of plants (Liang et al., 2018). Earlier investigations have exhibited that N metabolism was suppressed in plants exposed to Cd toxicity (Balestrasse et al., 2003; Gill et al., 2012; Wani et al., 2017; Shahid et al., 2019).

Consequently, a plausible action is indispensable to allay the harmful effects of Cd on plant metabolic events. In light of several reports, it is amply clear that plant growth regulators, both natural and synthetic, can effectively regulate the metabolic phenomena taking part in growth (Sheng et al., 2016; Asgher et al., 2017). Of these regulators, methyl jasmonate (MeJA) is one of the potential internal regulators involved in regulation of an array of physiobiochemical processes taking part in plant ontogeny (Mustafa et al., 2016; Yu et al., 2019). For instance, it is believed to be actively involved in transcriptional programming for improving tolerance to multiple stresses (Faghih et al., 2017; Ho et al., 2020). The involvement of MeJA in modulating the activities of some crucial antioxidant enzymes in most plants under heavy metal stress such as arsenic stress has been found in different plants such as oilseed rape (Farooq et al., 2018) and rice (Mousavi et al., 2020; Verma et al., 2020), as well as Cd stress in *Kandelia obovata* (Chen et al., 2014) and foxtail millet (Tian et al., 2017).

Nitric oxide (NO) is another prospective biostimulator that plays a critical role in a myriad of metabolic processes in plants exposed to heavy metal stress (Corpas, 2017; Terrón-Camero et al., 2019). NO can lessen the detrimental effects of oxidative injury by upraising the antioxidant systems, which can efficiently scavenge the ROS accrued under stress situations, or it may function as a vital signal in several key molecular processes (Hasanuzzaman et al., 2018; Sadhu et al., 2019). Many studies testify that NO functions as a signal in hormonal and environmental reactions in plants (Li et al., 2020; Sharma et al., 2020). Although MeJA and NO have been tested individually on plants under various heavy metals including Cd, the joint effect and crosstalk between both metabolites on alleviation of Cd stress on plants have not been yet elucidated. So, in the current study, the premier aim was to examine the effect of combined application of these two biostimulants and their crosstalk taking part in mitigation of Cd stress in wheat plants.

## Materials and Methods

### Plant Growth Under Varying Regimens

The investigation was performed under greenhouse conditions at 20–25°C and over 10°C, day and night temperatures, respectively, and with relative humidity 65–70%. Before initiation of the experiment, the wheat (*Triticum aestivum* L.) cv. “Pandas” seeds were treated with NaOCl (1% v/v solution) for sterilization. Each pot contained 5.0 L of perlite. At the beginning, 50 seeds were planted in each pot for germination, but thereafter only 35 plants were kept in each pot. During the growth season, the photoperiod was 11/13-h light–dark. Hoagland nutrient solution (NS; half strength) 0.1–1.0 L, based on plant size, was applied to each pot on alternate days during the discourse of the study. Additional details of the NS composition are given elsewhere (Steinberg et al., 2000). The pH of the NS was kept at 5.5. The layout of the experiment was a randomly completed block with three biological replications, each containing three pots. So, there were nine pots in each treatment.

**FIGURE 1:**
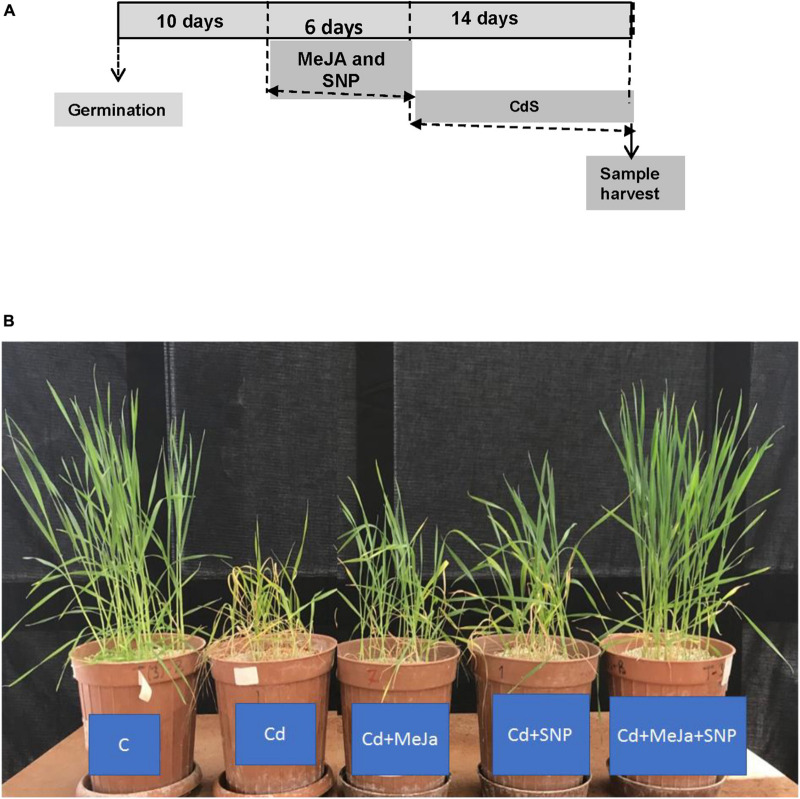
**(A)** Scheme of the treatments used to study the effect of cadmium (Cd) in wheat plants. The used concentration of each chemical was 100 μM Cd, 10 μM methyl jasmonate (MeJA) and 100 μM sodium nitroprusside (SNP) as nitric oxide donor (NO) and **(B)** effects of 10 μM MeJA and MeJA plus 100 μM SNP on the growth of wheat plants subjected to Cd. Photographs were taken at the end of the experiment.

Ten days after germination, different treatments were initiated. The wheat seedlings were exposed to Cd treatment (100 μM Cd) as cadmium chloride or without Cd treatment (control). These seedlings were further permitted to grow for 14 days. During this period, the wheat seedlings were sprayed every other day with 20 mL of deionized water per pot (control) or MeJA, sodium nitroprusside (SNP), or MeJA + SNP in 0.01% Tween-20 (Figure 1A). The control plants were well separated to avoid the spray of MeJA or SNP solution. Thereafter, the data of different parameters listed below were determined.

After determination of fresh weight, the shoot and root materials were separately kept at 75°C for 2 days in an oven and dry weights recorded.

### Determination of Cd Content, Biological Accumulation Coefficient, Biological Concentration Factor, and Translocation Factor

One container (containing 35 seedlings) per replication was chosen for chemical analysis. For the quantification of shoot and root Cd contents, digestion of dried samples was performed in HClO_4_:HNO_3_ solution (1:5, vol/vol) and then read with an inductively coupled plasma–optical emission spectrometry. The calculation of biological concentration factor (BCF), translocation factor (TF), and biological accumulation coefficient iological accumulation coefficient (BAC) was performed following the equations described by Malik et al. (2010). BCF represents Cd concentration ratio of roots to that of a growth medium. TF shows the ratio of Cd in plant shoots to that of roots. BAC shows the ratio of Cd in shoots to that in a growth medium.

### Quantification of Photosynthetic Pigments and Maximal Quantum Yield

Chlorophyll contents were quantified employing the procedure of Arnon (1949). After extraction of the leaf material in acetone (5 mL, 80%), the acetone (80%) solution was added to the extract to bring the final volume to 50 mL. The absorbance readings were taken at 480, 645, and 663 nm for carotenoids, chlorophyll a (Chl a), and chlorophyll b (Chl b), respectively.

The data for maximal quantum yield (*F*_*v*_/*F*_*m*_) were recorded with a portable Photosynthesis Yield Analyzer (Walz, Germany) on the leaves already subjected to dark for 30 min. The values of *F*_*v*_/*F*_*m*_ were worked out from different parameters obtained from the equipment such as minimal fluorescence (*F*_0_), maximal fluorescence (*F*_*m*_), and maximal variable fluorescence (*F*_*v*_ = *F*_*m*_ − *F*_0_) according to Maxwell and Johnson (2000).

### Determination of Relative Water Content, Proline, Glycine Betaine, and Total Soluble Sugars

The protocol highlighted by Barrs and Weatherley (1962) was pursued to estimate leaf relative water content (RWC). Fresh mass (FM) was quantified by weighing the cut leaves. Thereafter, the leaves were saturated in a Petri dish filled with water for 3 h to estimate the turgid mass (TM). Lastly, these materials were subjected to a drying oven at 80°C for 24 h to estimate dry mass (DM). The RWC was worked out using the following formula:

RWC(%)=[(FM-DM)/(TM-DM)]×100

Quantification of free proline (Pro) was performed according to Bates et al. (1973). To a proportion of 0.5 g fresh leaf, an aliquot of 10 mL of 3% sulfosalicylic acid was added, and then the material was centrifuged for 10 min at 3,000 *g.* Thereafter, an aliquot (2 mL) of the filtrate was treated appropriately with glacial acetic acid and acid ninhydrin. The mixture was subjected to 100°C for 1 h. After properly cooling the treated material, toluene (4 mL) was poured into it to separate free Pro. The OD was recorded at 520 nm.

The Grieve and Grattan (1983) method was followed for the quantification of glycine betaine (GB). The procedure employing the anthrone reagent was adopted to estimate total soluble sugars. The ethanol solution (80%) was used to extract the sample (0.1 g). Thereafter, the centrifugation of the mixture was performed for 10 min at relative centrifugal force (RCF) of 5,000. A supernatant of 0.5 mL was pipetted out, and 1 mL HCl (1N) was added to it. Then, it was subjected to a water bath at 100°C. An aliquot (4 mL) of 0.2% anthrone was poured into the sample mixture, and then it was kept in a water bath for another 10 min. The OD readings were noted at 620 nm (Fong et al., 1953).

### Quantification of Phytochelatins

The amount of GSH was subtracted from total non-protein thiols (NPTs) to obtain phytochelatin (PC) content. Fresh leaf tissue was macerated in sulfosalicylic acid (3%). Ellman’s reaction mixture contained 0.6 mM DTNB [5,5 o-ithiobis (2-nitrobenzoic acid)] and 5 mM EDTA. The quantification of NPT was noted at 412 nm (Ellman, 1959).

### Measurement of GSH and Ascorbic Acid

Fresh leaf tissue (500 mg) was homogenized in metaphosphoric acid buffer (3 mL, 5%) plus 1 mM EDTA. After centrifuging the extract for 12 min at RCF of 11,500 at 4°C, the reaction mixture was assayed for the appraisal of GSH and ascorbate.

The quantification of ascorbate was carried out according to Huang et al. (2005) by using potassium (K)–phosphate buffer (pH 7.0; 500 mM). The assay of reduced ascorbate was carried out in 0.1 M K–phosphate buffer (pH 7.0) and 0.5 U of ascorbate oxidase. The OD of all samples was noted at 265 nm.

Estimation of total AsA was performed following the extraction of each sample with 30 mM dithiothreitol. The content of dehydroascorbate (DHA) was calculated by deducting the content of reduced AsA from that of total AsA.

The assays of reduced GSH and glutathione disulfide (GSSG) were performed following Yu et al. (2003). To an aliquot of 0.4 mL of the sample extract, 0.6 mL of 500 mM K–phosphate buffer (pH 7.0) was added. The measurement of GSH was performed by the alterations in absorbance rate at 412 nm for NTB (2-nitro-5-thiobenzoic acid) produced by the reduction of DTNB. The GSSG concentration was worked out by eliminating GSH with 2-vinylpyridine (a derivatizing agent).

### Measurement of Oxidative Stress–Related Parameters

Quantification of leaf hydrogen peroxide (H_2_O_2_) was performed following the procedure optimized by Loreto and Velikova (2001). Fresh leaf material (0.5 g) was macerated in trichloroacetic acid (TCA, 3 mL of 1%). The centrifugation of homogenate was performed at 10,000 *g* for 10 min at 48°C. Thereafter, an aliquot of 0.75 mL of the homogenate was treated with 1 M KI (1.5 mL) and K buffer (10 mM, 0.75 mL). The OD values were recorded at 410 nm.

The estimation of leaf malondialdehyde (MDA) concentration was performed employing the method depicted by Weisany et al. (2012). The extraction of fresh leaf material (each 200 mg) was performed in TCA (5 mL of 0.1% wt/vol). The extract was centrifuged (12,000 *g*) for 5 min at 4°C. Then, by adding TCA (20%) to the extract, 4 mL of thiobarbituric acid (0.5%) was added to it. The treated sample material was warmed in a water bath at 90°C for 30 min. After bringing the temperature of the treated samples down to room temperature, their OD was read at 532 and 600 nm, respectively.

Electrolyte leakage (EL) was estimated by the method described by Dionisio-Sese and Tobita (1998). After washing fresh leaf tissue with deionized water to remove any contamination on the surface, leaf discs were excised. Then, to measure the initial electrolyte conductivity (EC1), those discs were kept in vials each containing 10 mL of deionized water for a day on a rotary shaker. Finally, those materials were incubated at 120°C for 20 min to measure the second electrolyte conductivity (EC2). To calculate the EL, the following formula was used:

EL(%)=(EC1/EC2)×100

### Analysis of Enzymatic Activities

Leaf tissue (500 mg) was macerated using 1 mL of 100 mM ice-cold K–phosphate buffer (pH 7.0) consisting of 1% polyvinylpyrrolidone (PVP). The well-ground material was centrifuged at 12,000 *g* for 15 min at 4°C. The extracted mixture was taken for the quantification of enzyme activities.

For the quantification of superoxide dismutase (SOD, EC 1.15.1.1) activity, the procedure of Van Rossum et al. (1997) was pursued. The mixture consisting of phosphate buffer (100 mM, pH 7.4), 10 mM of methionine, 1.0 mM EDTA, 75 μM of NBT, 50 μM of riboflavin, and 100 μL of the enzyme extract was kept under fluorescent light for 15 min. The OD of the treated samples was read at 560 nm.

The catalase (CAT, EC 1.11.1.6) activity was estimated following Chance and Maehly (1955).

The quantification of glutathione reductase (GR) activity was carried out as depicted by Hossain et al. (2010). The reaction solution consisted of K–phosphate buffer (100 mM, pH 7.8), 0.2 mM NADPH, 1 mM GSSG, 1 mM EDTA, and the enzyme extract in a final volume of 1 mL. The GSSG was added to start the reaction. The decline in optical density at 340 nm because of oxidation of NADPH was noted for 1 min.

The method of Hossain et al. (1984) was adopted to estimate the monodehydroascorbate reductase (MDHAR) activity. After properly treating the sample extract systematically with different chemicals listed in the method, its OD was read for 1 min at 340 nm.

The protocol of Nakano and Asada (1981) was employed to estimate the activity of dehydroascorbate reductase (DHAR). After treating the sample solution with a series of chemicals listed in the protocol, its OD was noted at 265 nm for 1 min.

The protocol of Hossain et al. (2006) was adopted to estimate the glutathione *S*-transferase (GST) activity. The reaction mixture consisted of 1.5 mM GSH, 100 mM Tris–HCl buffer (pH 6.5), 1 mM 1-chloro-2,4-dinitrobenzene, and enzyme solution in a final volume of 0.7 mL. The changes in absorbance were noted at 340 nm for 1 min. The activity was calculated using the extinction coefficient of 9.6 mM^–1^ cm^–1^.

The lipoxygenase (LOX) (EC: 1.12.11.12) activity was quantified using the protocol of Axelrod et al. (1981). Enzyme extract of 0.2 mL was treated with the reaction mixture (4 mL) comprising 50 mM Na-P buffer of pH 6.5 and 400 μM linoleic acid to start the reaction. The OD of all treated samples was recorded at 234 nm.

### Estimation of Total Free Amino Acids and Total Soluble Proteins

The aforementioned enzyme extract was also used to determine total amino acids following the ninhydrin method of Rosen (1957). The amount of total free amino acids was defined as μg glycine in 1 g of fresh material used. The procedure of Bradford (1976) was followed for assaying total soluble proteins in leaf tissues.

### Measurement of NR and NiR Activities

For the quantification of NR and NiR activities, the extraction of fresh leaf material (1:5, wt/vol) was carried out in a cold pestle and mortar using 100 mM potassium phosphate buffer (pH 7.5) consisting of 0.5% PVP, 2 mM EDTA, and 5 mM cysteine. After properly centrifuging the mixture, the filtrate was used for appraising the activities of NR and NiR.

The assay of NR activity was performed as outlined by Debouba et al. (2006). For the quantification of maximal NR activity, 1.4 mL of the sample mixture contained 100 mM potassium phosphate buffer (pH 7.5) that comprised 0.14 mM NADH, 7 mM KNO_3_, 10 mM MgCl_2_, and the enzyme extract. To commence the reaction, NADH was reacted with the sample extract. After that, it was incubated at 27°C for 30 min, and then 0.1 mL of 0.5 M zinc acetate was added to it, and the reaction mixture was subjected for 10 min to centrifugation at 3,000 *g.* The formation of NO_2_^–^ was quantified following the development of diazotization with 0.01% naphthylenediamine dihydrochloride and 1% sulfanilamide. Thereafter, its temperature was brought down to room temperature, and the absorbance values were noted at 540 nm. A standard calibration curve of NaNO_2_ was prepared to estimate the amount of NO_2_^–^ formed.

The activity of NiR was quantified as a decrease in the quantity of NO_2_^–^ in the reaction mixture following the procedure of Debouba et al. (2006). A 2.5 mL of the reaction mixture contained 2.3 mM methyl viologen, 0.1 M potassium phosphate buffer (pH 6.8), 0.4 mM NaNO_2_, and the enzyme extract. Sodium dithionite (4.3 mM) prepared in 0.1 M NaHCO_3_ was added to the reaction mixture to start the reaction. Then, it was subjected to 27°C for 30 min, and the reaction was terminated by shaking and boiling for 1 min. The ions of NO_2_^–^ left in the sample mixture were quantified at 540 nm.

### Determination of Glutamine Synthetase, Glutamate Dehydrogenase, and GOGAT Activities

For the quantification of GS and glutamate dehydrogenase (GDH) activities, fresh foliage tissue (1:5, wt/vol) was triturated in 50 mM Tris–HCl buffer (pH 7.6) comprising 1 mM MgCl_2_, 1 mM EDTA, 1 mM dithiothreitol, 10 mM β-mercaptoethanol, and 0.5% PVP. Then the extract was subjected to centrifugation at 20,000 *g* for 20 min, and the aliquot was taken for the quantification of the GS and NADH–GDH activities.

The activity of GS was quantified following the method of Agbaria et al. (1998). A 2.0 mL of the sample mixture consisted of 1 mM ADP, 50 mM Tris–HCl buffer (pH 7.2), 20 mM MgCl_2_, 50 mM glutamine, the enzyme extract, 20 mM sodium arsenate, and 13 mM hydroxylamine. To initiate the reaction, hydroxylamine was added to the sample mixture. Then the treated sample was subjected to 30°C for 30 min, and the reaction lasted by adding 3 mL of the solution containing 0.2 M FeCl_3_, 0.5 M HCl, and 0.24 M TCA. Then it was centrifuged at 3,000 *g* for 10 min, and the OD was read at 540 nm.

The assay of GDH activity was performed at 30°C by observing the oxidation of NADH at 340 nm by adopting the procedure of Groat and Vance (1981). For the determination of NADH–GDH activity, an aliquot of 2 mL of the sample mixture comprised 0.1 M Tris–HCl buffer (pH 8.0), 11 mM 2-oxoglutaric acid, the enzyme extract, 0.2 mM NADH, and 0.1 M NH_4_Cl.

For the quantification of GOGAT activity, fresh foliage tissue (1:5, wt/vol) was macerated in 50 mM potassium phosphate buffer (pH 7.5) consisting of 10 mM KCl, 2 mM EDTA, 1 mM phenylmethylsulfonyl fluoride, 14 mM β-mercaptoethanol, and ethylene glycol (3.58 M). Thereafter, the sample mixture was subjected for 20 min to centrifugation at 20,000 *g*, and the aliquot was taken for the quantification of NADH-GOGAT activity. The activity of NADH-GOGAT was quantified at 30°C by observing the oxidation of NADH at 340 nm following the protocol of Groat and Vance (1981); 2 mL of the sample mixture comprised 10 mM glutamine, 0.1 M potassium phosphate buffer (pH 7.5), 0.15 mM NADH, 5 mM 2-oxoglutaric acid, and the enzyme extract.

### Analysis of Total N, NO_3_^–^, and NH_4_^+^ in Wheat Shoot

The plant samples were subjected for 72 h to 70°C, and then total N in the samples was appraised using the Kjeldahl method described by Muñoz-Huerta et al. (2013).

For the quantification of NO_3_^–^ and NH_4_^+^, the extraction of fresh leaf material (1:10, wt/vol) was performed in redistilled water, and then the extract was heated well and filtered. The quantification of NO_3_^–^ was performed following the protocol of Cataldo et al. (1975). The reaction solution contained 0.2 mL of 5% salicylic acid prepared in concentrated H_2_SO_4_ and 0.1 mL of the filtrate. The treated sample mixture was kept for 15 min at room temperature, and then 1 mL of 4 M NaOH was mixed with it. After properly cooling the treated samples, they were read at 410 nm.

The quantification of NH_4_^+^ was performed using the Nessler reagent outlined by Molins-Legua et al. (2006). The reaction solution contained 0.1 mL of the Nessler reagent, 0.1 mL of the filtrate, 2.4 mL of redistilled water, and 0.01 mL of 10% K–Na tartrate. The OD was noted at 425 nm.

### Statistical Analysis

The data collected for all replicates of each parameter were subjected to a software SAS version 9.1 (SAS Institute Inc., Cary, NC, United States) for calculating analysis of variance. The figures or tables presented in the article present the means of each treatment along with standard error values. The significant differences among the treatment means were calculated using the Duncan multiple-range test at the 5% significance level.

## Results

### Phenotypic Appearance of the Wheat Plants

Figure 1B illustrates that the leaves of Cd-stressed wheat plants show clear-cut symptoms of chlorosis (yellowing). Plant height and leaf size of the plants also decreased prominently under Cd stress. The leaves of the plants supplemented with MeJa and MeJa + SNP did not show any symptoms of chlorosis and disorders mentioned earlier.

### Plant Growth, Photosynthesis-Related Parameters, and Translocation and Accumulation of Cd

**FIGURE 2:**
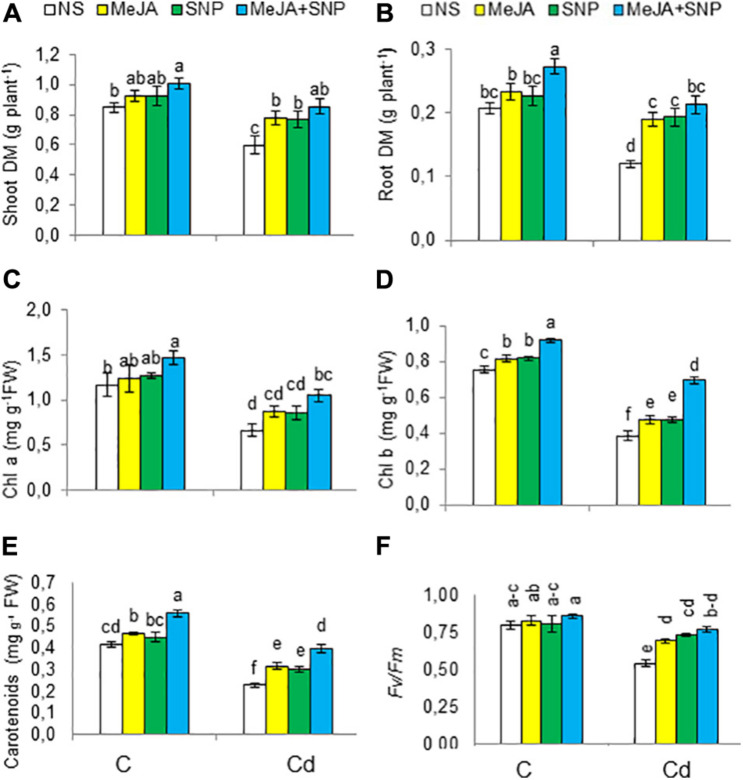
Shoot **(A)** and root **(B)** dry weight (DW), chlorophyll a **(C)**, chlorophyll b **(D)**, carotenoids **(E)** on fresh weight (FW) basis, and chlorophyll fluorescence parameters [*F*_*v*_/*F*_*m*_ **(F)**] in wheat plants grown under control (C) and cadmium (100 μM Cd) and sprayed with 10 μM methyl jasmonate (MeJA) or 100 μM sodium nitroprusside (SNP) alone or together (mean ± SE). Mean values carrying different letters within each attribute differ significantly (*P* ≤ 0.05) based on Duncan multiple-range test.

Cd significantly decreased the DM of different plant parts compared to the controls (Figures 2A,B). However, exogenously applied MeJA and SNP singly or jointly significantly mitigated the suppression in biomass. With respect to the controls, the aforementioned traits were found to be declined 29 and 41%, respectively, due to Cd, but a significant enhancement of 42 and 75% was recorded in these attributes, respectively, in the wheat plants treated with MeJA + SNP compared with those exposed to Cd toxicity only. Under non-stressed conditions, these chemicals also led to a marked rise in these attributes, attaining maximal values due to their combined supplementation. In view of these results, it could be safely inferred that SNP and MeJA actively participated in alleviation of Cd stress in the wheat plants. Compared with the controls, these parameters were increased by 19 and 29%, respectively, due to supplementation with MeJA + SNP.

The wheat seedlings exposed to high Cd regimen showed marked reductions in Chl a, Chl b, carotenoids, and photosystem II efficiency (*F*_*v*_/*F*_*m*_) by 44, 49, 43, and 33%, respectively (Figures 2C–F). Externally applied MeJA substantially enhanced Chl a, Chl b, carotenoids, and photosystem II efficiency (*F*_*v*_/*F*_*m*_), achieving the maximal values when both substances were applied together. Compared with the controls, these parameters were found to be raised by 37, 45, 70, and 43%, respectively, due to MeJA + SNP application.

**FIGURE 3:**
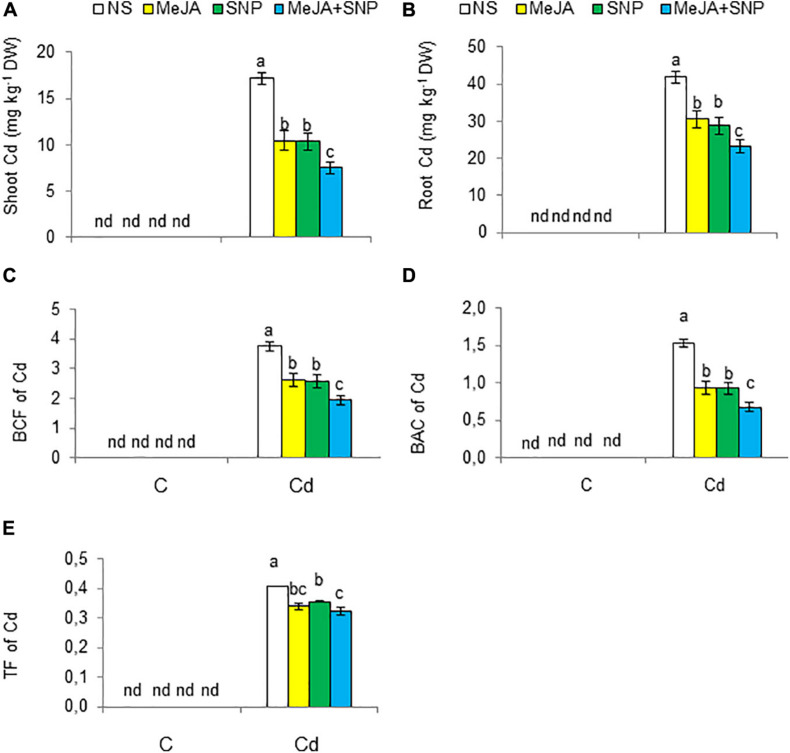
Shoot cadmium [Cd **(A)**], root Cd **(B)** on dry weight (DW) basis, the biological concentration factor [BCF **(C)**], biological accumulation coefficient [BAC **(D)**], and translocation factor [TF **(E)**] of Cd in wheat plants grown under control (C) and cadmium (100 μM Cd) and sprayed with 10 μM methyl jasmonate (MeJA) or 100 μM sodium nitroprusside (SNP) alone or together (mean ± SE). Mean values carrying different letters within each attribute differ significantly (*P* ≤ 0.05) based on Duncan multiple-range test. nd, not detected.

Cd was to be accumulated in both shoots and roots of the wheat plants grown under Cd toxicity (Figures 3A,B). The root Cd content was about 2.5-fold higher than that in the shoot. However, externally applied MeJA or SNP decreased the Cd content in roots by 27 and 31%, and in shoot by 39 and 41%, respectively, over those in the wheat seedlings exposed to Cd treatment without the supplementation of MeJA or SNP. Furthermore, the combined application (MeJA + SNP) led to a marked reducing effect on root and shoot Cd contents by 45 and 56%, respectively, over that in the Cd-stressed wheat seedlings.

The BCF, TF, and BAC of Cd from the growing medium to roots and shoots were also worked out. Cd increased BCF, TF, and BAC factors’ values (Figures 3C–E), but MeJA or SNP led to a substantial reduction in these values. Furthermore, the combination of both chemicals led to a further reduction in BCF (48%), TF (22%), and BAC (56%), suggesting a more effective protective defense against Cd toxicity.

### Modulation of RWC, Soluble Sugars, GB, and Pro Under Cd

**FIGURE 4:**
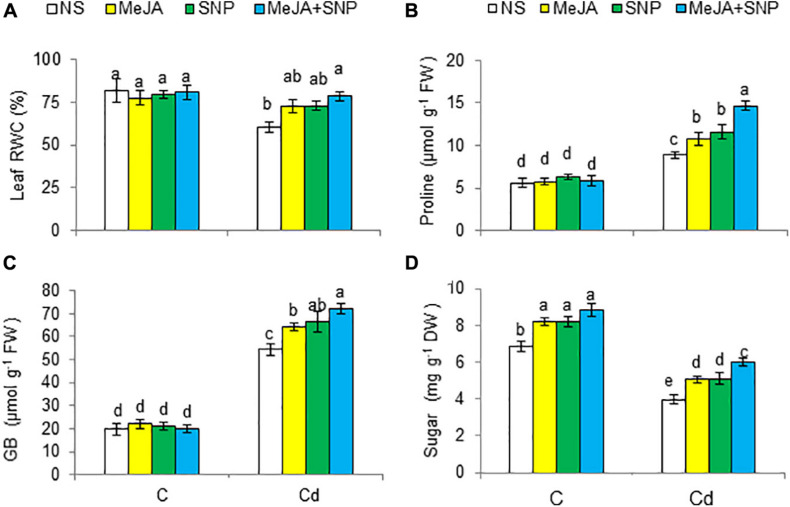
Leaf relative water content [RWC **(A)**], proline **(B)** glycine betaine [GB **(C)**] content on fresh weight (FW) basis, and sugar content **(D)** in wheat plants grown under control (C) and cadmium (100 μM Cd) and sprayed with 10 μM methyl jasmonate (MeJA) or 100 μM sodium nitroprusside (SNP) alone or together (mean ± SE). Mean values carrying different letters within each attribute differ significantly (*P* ≤ 0.05) based on Duncan multiple-range test.

With respect to controls, Cd reduced RWC by 27%; however, an alleviation of 20, 22, and 30% was attained due to MeJA, SNP, and MeJA + SNP application, respectively, relative to the controls (Figure 4A).

Cd raised the contents of Pro and GB by 59 and 172%, respectively, but it reduced total soluble sugars by 42% relative to the control (Figures 4B–D). The supplementation of MeJA and SNP singly and in combination enhanced the accumulation of these substances under Cd stress conditions. Individually, no marked difference was observed in the effectiveness of MeJA and SNP. With reference to that in the plants treated with Cd, percent increases in Pro, GB, and sugars were 67, 33, and 50%, respectively, in the wheat seedlings treated with MeJA + SNP.

### Improvement in PC Synthesis, GSH, and AsA Contents

**FIGURE 5:**
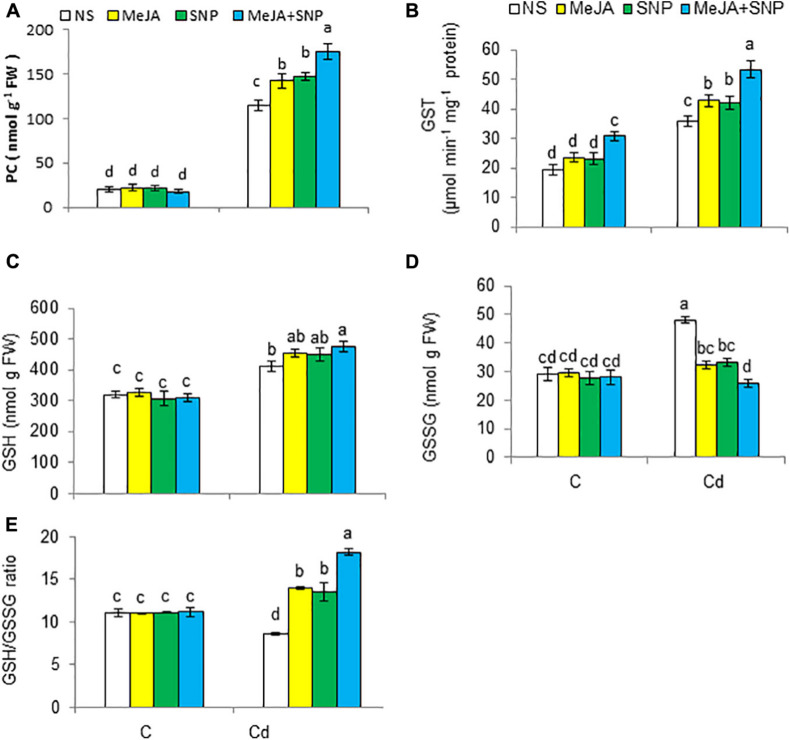
Phytochelatin [PC **(A)**], glutathione *S*-transferase [GST **(B)**], reduced glutathione [GSH **(C)**], oxidized glutathione [GSSG **(D)**] on fresh weight (FW) basis, and GSH/GSSG **(E)** in wheat plants grown under control (C) and cadmium (100 μM Cd) and sprayed with 10 μM methyl jasmonate (MeJA) or 100 μM sodium nitroprusside (SNP) alone or together (mean ± SE). Mean values carrying different letters within each attribute differ significantly (*P* ≤ 0.05) based on Duncan multiple-range test.

Cd toxicity boosted PC synthesis and the activity of GST by 5.9- and 1.7-fold, respectively (Figures 5A,B). Furthermore, Cd toxicity inverted the reduced levels of GSH and oxidized glutathione (GSSG) contents by 28 and 66%, respectively, but reduced the ratio of GSH/GSSG Figures 5C–E) over those in the controls. Externally applied MeJA or SNP together with Cd treatment led to further increases in these attributes, with the exception of reduction in GSSG. The combined effect of MeJA and SNP was more pronounced on those attributes compared with their individual effect. Furthermore, it can be concluded that MeJA and SNP treatments under Cd stress increased PC and GSH contents, which might have played an essential role in detoxification of Cd.

**FIGURE 6:**
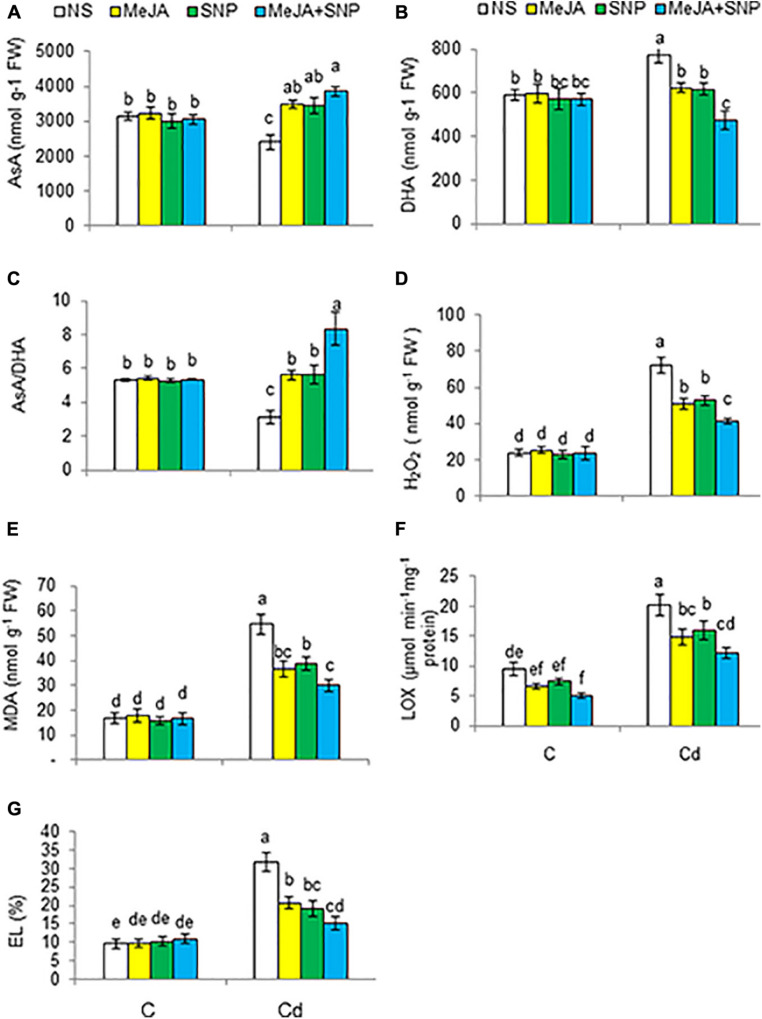
Ascorbate [AsA **(A)**], and dehydroascorbate [DHA **(B)**] on fresh weight (FW) basis, and AsA/DHA ratio **(C)**, hydrogen peroxide [H_2_O_2_ **(D)**], malondialdehyde [MDA **(E)**] on FW basis, lipoxygenase [LOX **(F)**], and electrolyte leakage [EL **(G)**] in wheat plants grown under control (C) and cadmium (100 μM Cd) and sprayed with 10 μM methyl jasmonate (MeJA) or 100 μM sodium nitroprusside (SNP) alone or together (mean ± SE). Mean values carrying different letters within each attribute differ significantly (*P* ≤ 0.05) based on Duncan multiple-range test.

Plants subjected to high Cd regimens exhibited a decline in AsA by 24%, but a rise in DHA content by 31% with reference to the controls (Figures 6A,B). Accordingly, the AsA/DHA ratio was reduced in the Cd-treated plants by 41% over the controls (Figure 6C). Externally supplied MeJA and SNP jointly with Cd treatment further improved AsA and AsA/DHA ratio, but a decline was observed in DHA content relative to those in the plants treated with Cd alone. Various doses did not affect the earlier-mentioned parameters under control conditions.

### Cd Induces Oxidative Stress

Cd treatment considerably improved the levels of H_2_O_2_ and MDA, EL, and the activity of LOX by 125, 210, 233, and 112%, respectively, over the controls (Figures 6D–G). These oxidative stress–related traits were found to be reduced due to the supplementation of MeJA or SNP. The combination of MeJA and SNP led to maximal reductions in the aforementioned parameters by 43, 46, 40, and 52%, respectively, with reference to the controls, i.e., only Cd-treated plants. Various treatments did not alter these parameters under control conditions.

### Modulation of the Antioxidant System

**FIGURE 7:**
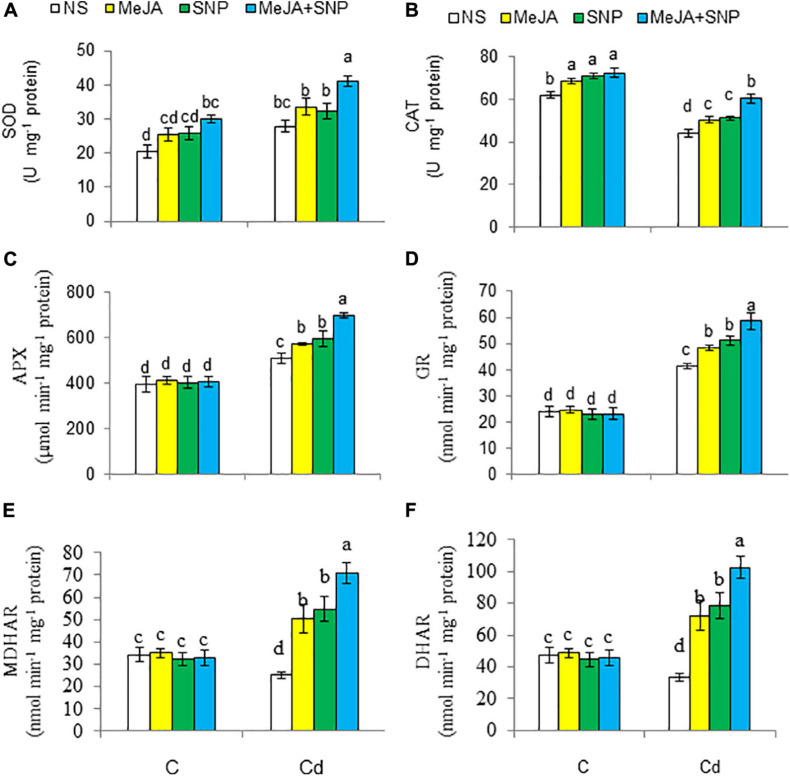
Activities of superoxide dismutase [SOD **(A)**], catalase [CAT **(B)**], ascorbate peroxidase [APX **(C)**], glutathione reductase [GR **(D)**], monodehydroascorbate reductase [MDHAR **(E)**], and dehydroascorbate reductase [DHAR **(F)**] in wheat plants grown under control (C) and cadmium (100 μM Cd) and sprayed with 10 μM methyl jasmonate (MeJA) or 100 μM sodium nitroprusside (SNP) alone or together (mean ± SE). Mean values carrying different letters within each attribute differ significantly (*P* ≤ 0.05) based on Duncan multiple-range test.

Acknowledgments The activities of enzymes of the antioxidant defense system are presented in Figures 7A–F. With respect to the controls, high Cd regimens boosted the activity of SOD (35%) and that of GR (71%), but it decreased CAT (29%), MDHAR (26%), and DHAR (30%). These enzyme activities were elevated in the wheat plants sprayed with MeJA or SNP, and the combined supply of the two chemicals was more pronounced on these enzyme activities over those in the Cd-stressed wheat plants. Under the control growing conditions, the wheat plants supplemented with MeJA and SNP showed a marked elevation in the activities of CAT and SOD, but they showed insignificant alterations in the activities of GR, MDHAR, and DHAR.

### Improvement in N Metabolism Under Cd Stress

**FIGURE 8:**
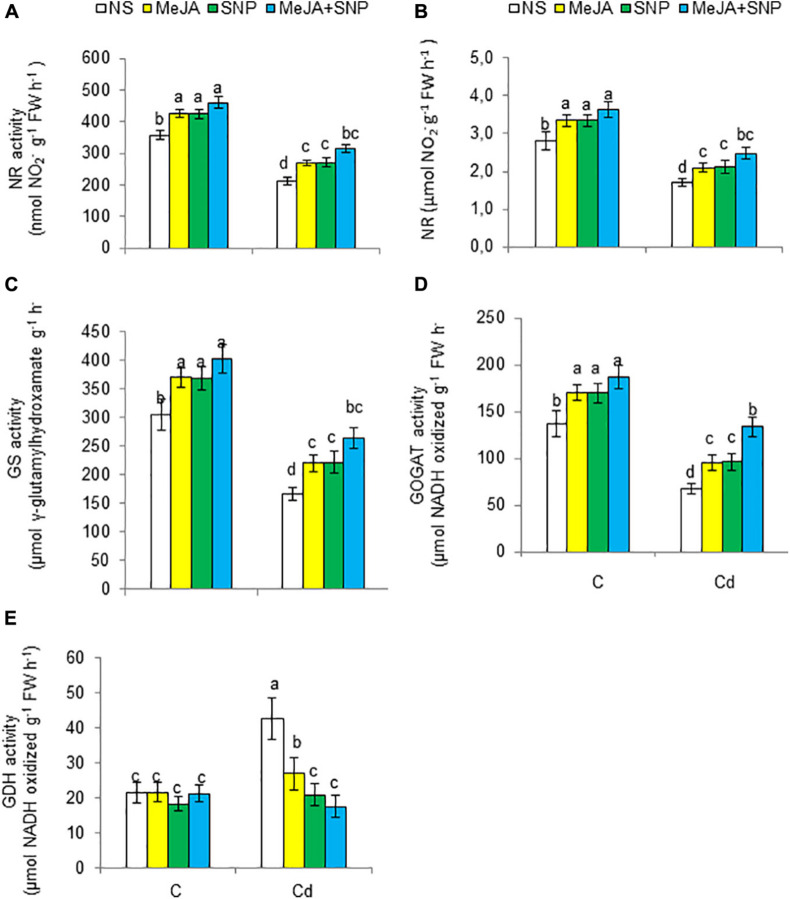
Activities of nitrate reductase [NR **(A)**], nitrite reductase [NiR **(B)**], glutamine synthetase [GS **(C)**], glutamate synthase [GOGAT **(D)**], and glutamate dehydrogenase [GDH **(E)**] on fresh weight (FW) basis in wheat plants grown under control (C) and cadmium (100 μM Cd) and sprayed with 10 μM methyl jasmonate (MeJA) or 100 μM sodium nitroprusside (SNP) alone or together (mean ± SE). Mean values carrying different letters within each attribute differ significantly (*P* ≤ 0.05) based on Duncan multiple-range test.

High Cd regimens led to marked decreases in N metabolism–related enzymes such as NR, NiR, GS, and glutamate synthase (GOGAT) by 41, 39, 46, and 50%, respectively, but it increased the activity of GDH by 49% (Figures 8A–E). However, the wheat plants sprayed with MeJA or SNP alone showed a rise in the activities of NR, NiR, GS, and GOGAT and a decline in GDH. Furthermore, Cd-stressed plants sprayed with both substances together exhibited increases of 49, 44, 59, and 97% for NR, NiR, GS, and GOGAT, respectively, but a decrease in GDH by 58% over that in the Cd-stressed plants.

**FIGURE 9:**
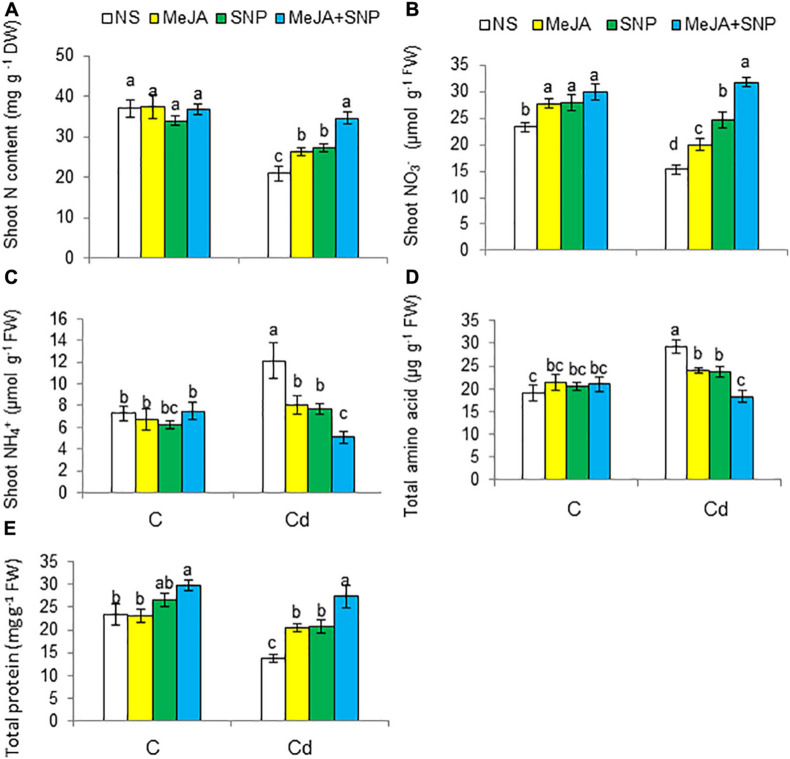
Shoot total nitrogen [N **(A)**] on dry weight (DW) basis, shoot nitrate [NO_3_^–^ **(B)**], shoot ammonium [NH_4_^+^ **(C)**], total amino acid **(D)**, and total protein **(E)** contents on fresh weight (FW) basis in wheat plants grown under control (C) and cadmium (100 μM Cd) and sprayed with 10 μM methyl jasmonate (MeJA) or 100 μM sodium nitroprusside (SNP) alone or together (mean ± SE). Mean values carrying different letters within each attribute differ significantly (*P* ≤ 0.05) based on Duncan multiple-range test.

The contents of total N and NO_3_^–^ decreased by 43 and 35%, respectively, but that of NH_4_^+^ increased by 71% in the Cd-stressed wheat plants compared with the controls (Figures 9A–C). The wheat plants fed with MeJA or SNP exhibited a rise in total N and NO_3_^–^, a decline in NH_4_^+^ under Cd stress, a maximal increase of 57 and 107% for N and NO_3_^–^, respectively, but a decrease of 56% for NH_4_^+^ was observed in the plants sprayed with MeJA and SNP jointly.

Relative to the controls, Cd stress enhanced total amino acid content by 53%, but reduced total protein content by 40% in the leaves of the wheat plants (Figures 9D,E). The application of MeJA or SNP led to a decline in total amino acids, but a rise in total proteins under Cd stress. A maximal decrease in total amino acids by 38% and an increase in total proteins by 108% were observed in the plants fed with MeJA and SNP together with reference to the controls, i.e., plants treated with Cd only.

## Discussion

### Improvement in Wheat Plant Growth Under Cd Stress

Contaminations of heavy metals detrimentally influence the plant growth as similarly observed in other stresses (Ahanger et al., 2020). Cd-induced reduction in plant growth was found in our study, which is similar to that reported by Anjum et al. (2016) in maize, and Afzal et al. (2019) in rice. The most likely reason of reduced plant growth due to Cd could have been due to reduced uptake of water and mineral elements (Nahar et al., 2016) and reduced metabolism of sugars and photosynthetic efficiency (Zaid and Mohammad, 2018). The supply of MeJA and SNP mitigated Cd-induced reduced plant growth in our study. Especially, the joint supply of MeJA and SNP was more in effect in promoting growth of Cd-stressed wheat seedlings, as analogously recorded in mustard (Per et al., 2016) and *Mentha arvensis* (Zaid and Mohammad, 2018) in which MeJA increased plant biomass under Cd stress. In some other studies, SNP application was reported to be efficient in promoting growth of Cd-stressed plants, e.g., tall fescue (Zhuo et al., 2017) and *Catharanthus roseus* (Nabaei and Amooaghaie, 2020). The beneficial effect of MeJA and SNP on mitigation of Cd-induced suppression in growth of the wheat plants might have been linked with improved *F*_*v*_/*F*_*m*_ and leaf chlorophyll contents (Per et al., 2016; Kaya et al., 2019).

### Reduction in Shoot and Root Cd Contents in Wheat Under Cd

Cd is easily absorbed by plants growing in soils having toxic levels of Cd and is mostly accumulated in the roots, and relatively less quantity is transported to the shoot of plants (Nahar et al., 2016). Cell membrane and wall are the major blockers against influx of Cd to the cell (Zhang et al., 2017). The entrance of Cd to the cell can also be reduced by PCs (Uraguchi et al., 2017). In our experiment, Cd caused accumulation and a substantial elevation in Cd in the plant tissues, especially higher Cd content in the roots than that in the shoots, which in turn led to a higher decrease in the growth of roots than that in the shoot, as previously recorded in wheat (Qin et al., 2018). So, a variety of approaches have been tested to alleviate the detrimental impact of metals by inhibiting their transport to plants (Koźmińska et al., 2018). For instance, the use of externally supplied plant hormones has been focused on by researchers to relieve the damaging effects of heavy metals (Sytar et al., 2019). In the current experimentation, the potential impact of the application of MeJA and SNP singly or jointly in allaying the injurious effects of Cd stress on wheat plants was assessed. Both MeJA and SNP led to a marked reduction in Cd absorption by the roots. Furthermore, MeJA and SNP lowered the transfer of Cd from the roots to the aboveground plant parts. The joint effect of MeJA and SNP was more effective on reducing the root uptake of Cd and Cd translocation from roots to shoot with respect to its single application. Our results are in line with those reported in other studies. For example, externally supplied MejA decreased Cd content in foxtail millet (Tian et al., 2017) and wheat plants (Alikhani and Abbaspour, 2019). Furthermore, the reducing effect of SNP on shoot and root Cd has also been observed in *C. roseus* (Nabaei and Amooaghaie, 2020) and rice (Singh P. et al., 2020). The reduced absorption of Cd may be because of the development of a metal Cd–NO complex as suggested by Singh P. et al. (2020), but this needs to be further confirmed. Alternatively, it has been suggested that NO might defend plants against membrane injury, thereby maintaining their regular metabolic processes, as a minor portion of Cd moves to the shoot cells (Dong et al., 2016). Furthermore, externally applied MeJA and SNP reduced Cd BCF, transport factor (TF) from roots to shoots, and biological accumulation concentration (BAC); this shows the crucial controlling roles of MeJA and SNP in advancing tolerance to Cd by decreasing the Cd transportation and accumulation as similarly suggested by Nahar et al. (2016).

### Photosynthesis-Related Parameters in Wheat Under Cd Stress

The exogenous application of MeJA together with SNP showed more encouraging effect on the contents of chlorophyll and carotenoids, as well as *F*_*v*_/*F*_*m*_ under control and Cd stress. Some previous reports also show that MeJA or SNP improves photosynthesis-related parameters, e.g., in mustard (Per et al., 2016) and *M. arvensis* (Zaid and Mohammad, 2018), in which MeJA was reported to improve the aforementioned attributes. The beneficial effect of NO on these parameters was also testified in cucumber (Gong et al., 2017) and ryegrass (Chen et al., 2018) under Cd. The possible reason of the constructive role of MeJA and SNP on photosynthesis-related parameters under Cd stress could be linked to reduced chlorophyll breakdown and reduced ROS together with enhanced antioxidant enzymes’ activities. Gong et al. (2017) suggested that NO strongly protects photosynthetic apparatus from Cd. Per et al. (2016) observed that MeJA enhanced the synthesis of GSH, thereby improving chlorophyll synthesis as similarly observed in our experiment. Reduced chlorophyll degradation, improved GSH contents, and the rise in the antioxidant system due to MeJA or SNP allow the plants to thrive well under Cd stress. In our study, plants treated with MeJA and SNP also exhibited lower contents of H_2_O_2_ and MDA, and higher chlorophyll content than those in the Cd-stressed plants. Earlier investigations have noted a beneficial effect of MeJA in wheat (Alikhani and Abbaspour, 2019) and that of NO in rice (Hsu and Kao, 2004) and tomato (Ahmad et al., 2018) under Cd stress. There seems to be no investigation reporting the joint effects of MeJA and SNP on chlorophyll synthesis in the current literature.

### Water Relations and Osmolytes in Wheat Under Cd Stress

The application of MeJA and SNP leads to higher accumulation of osmotic substances, which can improve stress tolerance by enhancing water status in the cells of plants (Arasimowicz-Jelonek et al., 2009; Sadeghipour, 2018). Like other osmotic substances, both Pro and GB participate in alleviation of a stress through osmotic adjustment in plants (Bhuiyan et al., 2019; Zhang and Yang, 2019). Furthermore, Cd stress elevated the accumulation of Pro (Zouari et al., 2016) and GB (Jan et al., 2018). In our study, MeJA and NO application increased the production of GB and Pro; this obviously shows that MeJA and NO play a protecting function in Cd tolerance. Some previous investigations also exhibit that exogenously supplied MeJA augmented Pro and GB contents as observed in *M. arvensis* (Zaid and Mohammad, 2018), and that of NO increased Pro in alfalfa (Su et al., 2018), and GB and Pro in tomato (Ahmad et al., 2018) under Cd stress. Furthermore, our observations suggest that MeJA- and NO-induced enhanced generation of Pro could have been due to the modulation of Pro metabolism, as suggested for the effect of NO by Ahmad et al. (2018) under Cd stress. Increased Pro and GB in MeJA- or SNP-applied plants led to improvement in leaf RWC, possibly due to improvement in hydraulic conductivity, as suggested by Ahmad et al. (2018), who reported that NO improved Pro, which in turn enhanced the hydraulic conductivity in tomato. Moreover, Cd stress is believed to inhibit water uptake (Naeem et al., 2019) due to reduced root hydraulic conductance, which may lead to a substantial decrease in cellular turgor, thereby leading to reduced RWC.

### Upregulation of Cd Detoxification Process in Wheat

The metabolites, such as GSH, PCs, and GST activity are straightly involved in the detoxification of Cd (Li et al., 2018). Metals can be bound to the thiol (-SH) group of GSH (Baig et al., 2019), and in this way, GSH can sequester a metal and transfer it to the vacuole as a precursor of PCs (Ramakrishna and Gill, 2018). Similarly, PCs can efficiently bind to Cd as a chelating agent (Rahimzadeh et al., 2017). Heavy metal stress increases PC contents, which are believed to have a potential role in the detoxification of Cd (Uraguchi et al., 2017). This shows that PCs and GSH together detoxify Cd in plants. Plants under Cd stress showed a rise in GSH, which may detoxify Cd, and it is converted to GSSG; this could have been the reason of GSSG being higher in the Cd-treated wheat plants than that in the controls. High GSSG together with decreased GSH/GSSG ratio indicates an oxidative injury induced by Cd (Singh S. et al., 2020). The application of SNP and MeJA inverted the GSH/GSSG ratio and GSH content by rising the GSH concentration and GSH/GSSG ratio; our findings are also in line with those of an earlier report of Per et al. (2016) wherein MeJA increased GSH in mustard under Cd stress. Nahar et al. (2016) also testified restoration effect of SNP on GSH and GSH/GSSG ratio in Cd-treated mung bean. The PC synthesis increased in Cd-stressed wheat seedlings, and MeJA and SNP further increased the PC concentration in the Cd-fed plants with reference to the plants treated with Cd only. This obviously shows that MeJA and SNP alone or in combination participate in activating the PC biosynthesis, which in turn leads to the chelation of Cd. Hu et al. (2019) also reported that NO synthesis improved chelation of Cd in *Sedum alfredii*. Moreover, Alikhani and Abbaspour (2019) reported the MeJA showed a beneficial effect on the chelation of Cd in wheat. The combined effect of MeJA and SNP was more pronounced in terms of increasing the PC content. There seems to be no study in the literature on the combined effect of these two metabolites on PC synthesis in Cd-stressed plants.

### Reversal of Oxidative Injuries Under Cd Stress

The application of MeJA and SNP substantially suppressed the contents of H_2_O_2_ and MDA, the activity of LOX, and EL in the wheat plants under Cd stress. Previous reports also exhibit that Cd elevated the aforementioned oxidative stress parameters, particularly in tomato (Alyemeni et al., 2018) and *Glycine max* (Molina et al., 2020) plants. Overaccumulation of H_2_O_2_ can damage crucial processes of cells including photosynthesis by decreasing the strength of organelle ultrastructure’s (Smirnoff and Arnaud, 2019). Furthermore, high H_2_O_2_ accumulation in plants under a stress results in enhanced injury to lipids and proteins, which in turn affects their integrity and effectiveness (Palma et al., 2019); this was also obvious in our investigation in terms of enhanced MDA and EL. Cd-induced ROS accumulation leads to the generation of LOX (Kaya et al., 2019), which is known as an indicator of considerable injury to lipids. In our study, externally applied MeJA and SNP decreased the LOX activity in Cd-stressed wheat plants. NO has been shown to reduce LOX activity under Cd stress (Nahar et al., 2016). Reduced membrane leakage due to the application of MeJA and SNP might have been due to improved antioxidant activity.

### Enhancement in Antioxidants in Wheat Under Cd Stress

The supply of MeJA and SNP-induced reversal of oxidative injury under Cd stress might have been due to upregulation of the antioxidant system. Accordingly, MeJA and SNP supplementation substantially elevated the activities of antioxidant enzymes such as CAT, SOD, and the AsA–GSH cycle–related enzymes under Cd treatment. Various antioxidant enzymes perform specific functions in different parts of the cell (Caverzan et al., 2016). SOD is pervasive in the scavenging of superoxide, but CAT eliminates H_2_O_2_ in the cytosol (Azarabadi et al., 2017). Furthermore, AsA–GSH cycle enzymes and components of the redox system, AsA and GSH, can scavenge H_2_O_2_, thereby sustaining electron transport (Caverzan et al., 2019). Previous results have reported that application of MeJA augmented the activities of SOD and GR in mustard (Per et al., 2016) and *Arabidopsis* (Lei et al., 2019) under Cd stress. The MeJA-induced enhanced activities of the antioxidant enzymes might have been due to the interaction of MeJA with H_2_O_2_. This has also been suggested by Zaid and Mohammad (2018). MeJA and SNP enhanced the GR activity, which can promptly remove H_2_O_2_ through the AsA–GSH cycle system, thereby resulting in allaying Cd-induced oxidative injury in the wheat seedlings. Earlier investigations have suggested that MeJA decreases oxidative stress due to Cd toxicity by elevating the GSH levels and antioxidant enzyme activities, thus reducing the levels of MDA and H_2_O_2_ in plants under high Cd regimens (Zaid and Mohammad, 2018; Lei et al., 2019), similar to that found in the current research. Our results also indicate that NO elevated the SOD, CAT, and AsA–GSH cycle–related enzymes’ activities, as earlier recorded in wheat (Zhao et al., 2016) and tomato (Ahmad et al., 2018), but no studies reporting the joint effect of MeJA and SNP are available. The enhanced ASA-GSH cycle–related enzymes’ activities due to MeJA and SNP application may have provided higher defense to cellular organelles and metabolic processes against the harmful effects caused by Cd stress.

Cd suppressed AsA levels and enhanced those of DHA in the wheat seedlings, as earlier observed in *Brassica napus* (Maresca et al., 2017). However, addition of MeJA and SNP with Cd reduced DHA content and elevated that of AsA by enhancing the MDHAR and DHAR activities, which elevated AsA/DHA ratio under Cd stress. Nahar et al. (2016) also observed increased DHAR activity in mung bean treated with NO under Cd stress.

### Upregulation of N Metabolism in Wheat Under Cd Stress

N metabolism can be disturbed by high Cd regimens (Khan et al., 2015; Ahanger et al., 2020). For example, Cd stress reduced NR activity and total N and NO_3_^–^ contents, but it increased NH_4_^+^ content in the wheat plants. Analogous findings can be seen elsewhere, showing that Cd reduced the NR activity and N content in plants (Khan et al., 2015; Ahanger et al., 2020). Plants use NO_3_^–^ as an N source (Liu et al., 2018); NR acts as a main catalytic enzyme in the reduction of NO_3_ to NO_2_ in plant cells (Yoneyama and Suzuki, 2019). Then, NO_2_ is converted to NH_4_^+^ by NiR activity (Balotf et al., 2016). Decreased NO_3_ content due to Cd in the wheat plants might be linked with reduced transpiration due to Cd stress; this may have led to decreased NO_3_ transport from the root to the above parts of plants through xylem (Singh and Prasad, 2017). Alternatively, overaccumulation of ROS due to Cd led to enhanced cell membrane permeability, thereby reducing the absorption of NO_3_^–^ by the root cells (Ramakrishna and Gill, 2018). Moreover, reduced absorption of NO_3_ and NR activity, as observed in the wheat plants, has also been observed by Singh and Prasad (2017) in Cd-stressed tomato plants. However, augmentation of NH_4_^+^ content in the Cd-stressed wheat plants may have occurred because of limitation in the assimilation of ammonia (Khan et al., 2016; Yang et al., 2019). Overaccumulation of NH_4_^+^ is harmful to the plant cells (Wang et al., 2019), and so plants possess a strategy through the GS/GOGAT pathway or GDH pathway, which are other paths for assimilating NH_4_^+^ so as to lower the harmful effects due to the accumulation of ammonia, particularly when the activity of GS is diminished (Aly et al., 2018). NH_4_^+^ is transformed into an organic substance mainly by the GS/GOGAT pathway (Ashraf et al., 2018). The findings of our experimentation exhibited that reduced GS and GOGAT activities in the wheat plants under Cd stress could be linked to the disruption in NH_4_^+^ assimilation, which is evident by reduced N and protein contents and increased NH_4_^+^ content. However, Cd-induced increased GDH activity might have reduced the activities of GS and GOGAT. Enhanced GDH activity could not be high enough to maintain NH_4_^+^ assimilation; this was evident as enhanced NH_4_^+^ content along with reduced growth occurred in the Cd-stressed wheat plants. However, enhanced GDH activity is believed to participate in lessening NH_4_^+^ accumulation and glutamate production for the synthesis of defensive substances (Xiaochuang et al., 2020).

The exogenous supply of MeJA and SNP markedly augmented the NR activity, total N, and NO_3_ and NO_2_ contents, as well as reduced the NH_4_^+^ content due to rapid use of NH_4_^+^ to produce amino acids via enhanced GS and GOGAT activities. This led to enhanced utilization of N in chlorophyll synthesis, thereby increasing plant growth in the wheat plants under Cd stress. NR regulates the rate-restricting reaction in N metabolism, which in turn mediates the crucial physiological processes, e.g., amino acids and secondary metabolites containing N (Ahanger et al., 2017; Chamizo-Ampudia et al., 2017). Similarly, Zaid and Mohammad (2018) noted that MeJA increased the NR activity and N content. Increased uptake and assimilation of NO_3_^–^ lead to increased conversion of available N to amino acids (Hachiya and Sakakibara, 2017). Furthermore, elevated NR activity leads to enhanced N assimilation (Alt et al., 2017), which in turn increases stress tolerance by probably increasing the protein synthesis. Furthermore, application of MeJA and SNP reduced the GDH activity in the Cd-stressed wheat plants, suggesting that this enzyme enhances the main pathway of NH_4_^+^ assimilation by regulating the GS/GOGAT cycle under Cd stress. Moreover, MeJA and SNP-induced mitigation of Cd toxicity is most likely due to enhanced content of proteins.

## Conclusion

Cd impaired growth, water relations, N metabolism, and AsA–GSH cycle. The combined application of MeJA and SNP showed promising results in terms of improving plant growth and physiology and N metabolism, upregulating the AsA–GSH cycle–related enzymes’ activities, and reducing Cd content in wheat plants, suggesting a possible interactive role of the two compounds in alleviating Cd-toxicity in wheat plants. This work further suggests an ecofriendly approach for mitigation of heavy metal toxicity in food cereals.

## Data Availability Statement

The raw data supporting the conclusions of this article will be made available by the authors, without undue reservation.

## Author Contributions

CK, MA, and PA designed the experimentation. CK performed the experiments and generated the data. MA, HD, and AN analyzed the data. CK and MA jointly wrote up the manuscript. MA and PA thoroughly edited the entire manuscript. All authors contributed to the article and approved the submitted version.

## Conflict of Interest

The authors declare that the research was conducted in the absence of any commercial or financial relationships that could be construed as a potential conflict of interest.

## Publisher’s Note

All claims expressed in this article are solely those of the authors and do not necessarily represent those of their affiliated organizations, or those of the publisher, the editors and the reviewers. Any product that may be evaluated in this article, or claim that may be made by its manufacturer, is not guaranteed or endorsed by the publisher.
